# Analysis of the treatment and prognosis of 266 cases of extranodal natural killer/T-cell lymphoma, nasal type in a single medical center

**DOI:** 10.3389/fonc.2024.1388564

**Published:** 2024-04-03

**Authors:** Lei Yang, Liqiang Wei, Xin Li, Jia Cong, Jin Ye, Na Yao, Jing Yang, Liang Wang, Jingwen Wang

**Affiliations:** Department of Hematology, Beijing Tongren Hospital, Capital Medical University, Beijing, China

**Keywords:** extranodal NK/T-cell lymphoma, asparaginase, survival rates, prognostic factors, therapeutic outcomes

## Abstract

**Objective:**

To assess the impact of different treatment strategies and risk factors on the prognosis of patients with extranodal NK/T-cell lymphoma, nasal type (ENKTL) in a single medical center.

**Methods and analysis:**

The clinical features of 266 patients with ENKTL were retrospectively analyzed, among whom those in stages I and II received sandwich therapy, while those in stages III and IV underwent chemotherapy plus autologous hematopoietic stem cell transplantation. The Kaplan–Meier curves, univariate and multivariate Cox regression analyses were employed for survival and prognosis analysis. Statistical significance was set at P<0.05.

**Results:**

Following treatment, the post-intervention outcomes demonstrated a complete remission (CR) rate of 71.05% and a partial remission (PR) rate of 3.76%. The 5-year progression-free survival (PFS) and overall survival (OS) rates were 70.4% and 70.9%, respectively. In addition, the PFS for patients in stage I/II was 79.8%, with an OS of 81.1%, whereas for those in stage III/IV, the PFS was 41.7% and the OS was 40.9%. Notably, the achievement of CR immediately after treatment was an independent prognostic factor (P<0.001). Patients in stage I/II depicted a favorable 5-year OS rate, while those in stage III/IV manifested a less favorable prognosis.

**Conclusion:**

Stages of the disease and whether CR was achieved following treatment are important factors determining the survival and prognosis of patients with ENKTL. Further researches focusing on disease onset and mechanisms of drug resistance will contribute to better management of ENKTL.

## Introduction

Extranodal natural killer/T-cell lymphoma, nasal type (ENKTL) is a subtype of non-Hodgkin’s lymphoma (1), derived from natural killer (NK) cells or T cells, with its incidence evidently associated with Epstein-Barr (EB) virus infection. ENKTL is a rare malignancy, particularly in Europe and North America. However, its incidence is relatively higher in Asia and Latin America, constituting 5 to 15% of non-Hodgkin’s lymphomas (2, 3). This disease is characterized by an aggressive course, nasal obstruction, nasopharyngeal ulcer, and tissue necrosis. Moreover, the majority of patients present with localized lesions, primarily occurring in the nasal cavity, nasopharynx, paranasal sinuses, oropharynx, and even in the upper respiratory and digestive tracts. A few cases display a progressive phenotype involving multiple organs, including the skin, adrenal gland, liver, spleen, and bone marrow (4). Patients in the early stage of ENKTL are sensitive to radiotherapy and exhibit a favorable prognosis (5, 6). However, for patients in advanced stages, the long-term recurrence rate after radiotherapy alone is high. In addition, the disease demonstrates insensitivity to traditional anthracycline-containing chemotherapy attributed to multidrug resistance, resulting in a poor prognosis. Thus, a regimen incorporating asparaginase with substantial clinical efficacy and safety has been developed, consequently becoming the first-line treatment for ENKTL (7, 8). However, owing to the rarity of this disease, few large-scale prospective, randomized, controlled clinical studies have been conducted. Therefore, the current treatment scheme still remains controversial, and a standardized treatment model is not yet established. In this study, we conducted a retrospective analysis of 266 patients with newly diagnosed ENKTL between May 2009 and February 2021 in the Department of Hematology, Beijing Tongren Hospital, with whom three chemotherapy schemes containing asparaginase were administered for comprehensive treatment. The aim of this study is to provide insights and guidelines for the clinical treatment of ENKTL disease.

## Materials and methods

### Patients and methods

**Figure 1:**
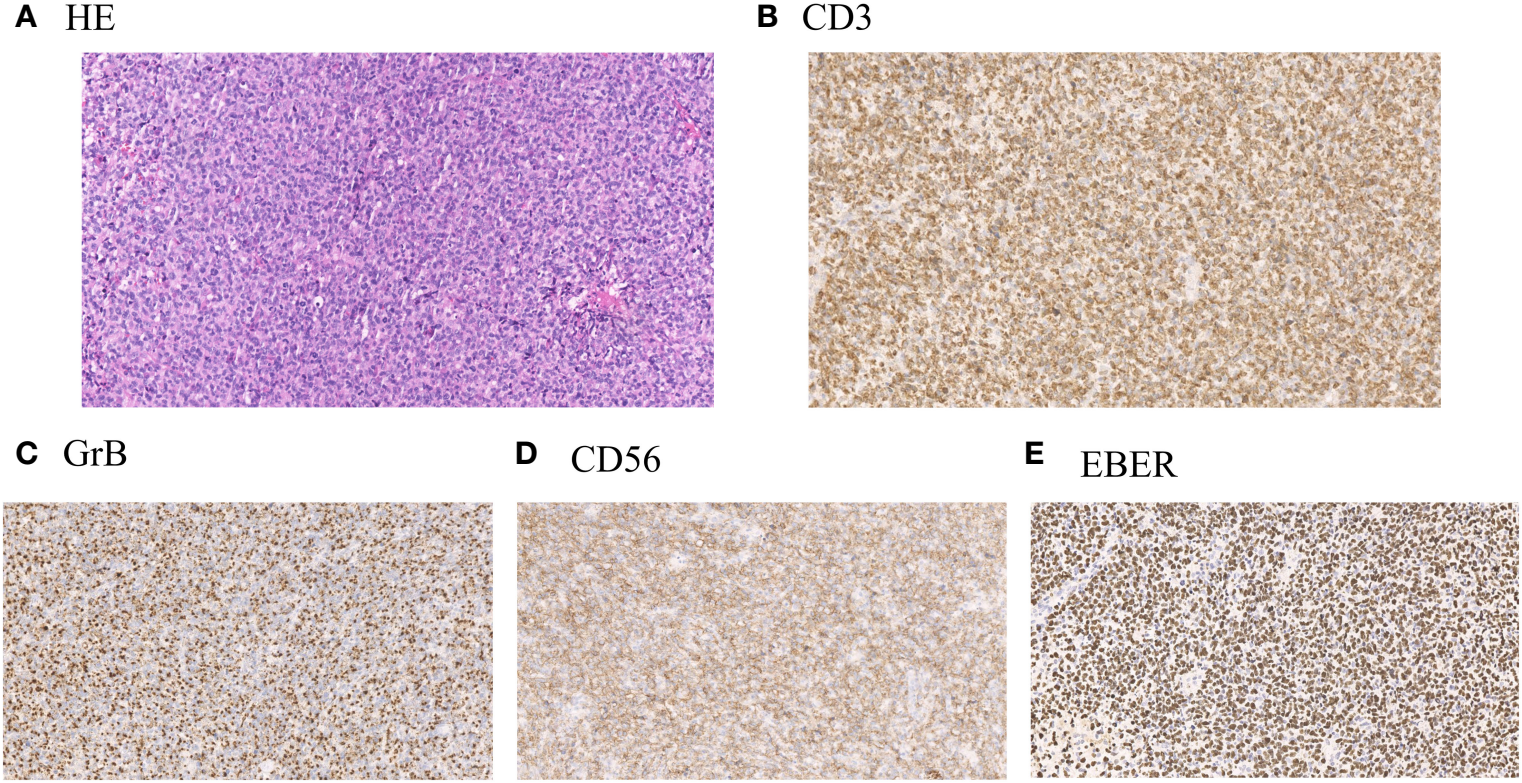
**(A)** Representative hematoxylin and eosin stain (H&E) of lymphoma samples obtained from patients with ENKTL. **(B)** Representative immunohistochemical staining of positive expression of CD3 in lymphoma samples from patients with ENKTL. **(C)** Representative immunohistochemical staining of positive expression of Granzyme B (GrB) in lymphoma samples from patients with ENKTL. **(D)** Representative immunohistochemical staining of positive expression of CD56 in lymphoma samples from patients with ENKTL. **(E)** Representative immunohistochemical staining of positive expression of Epstein–Barr virus–encoded small RNA (EBER) in lymphoma samples from patients with ENKTL.

The clinical data of 266 cases of ENKTL originating from the upper respiratory and digestive tracts, diagnosed between May 2009 and February 2021 in the Department of Hematology, Beijing Tongren Hospital, were retrospectively analyzed. After tissue biopsy, the pathological types were classified according to the 2016 World Health Organization (WHO) Classification Standard for Hematopoietic and Lymphatic Tumors. The diagnosis was performed by the pathologists at our hospital through histological and immunohistochemical analysis. Lymphoma cells frequently show angiocentricity and angiodestruction, resulting in zonal necrosis. Morphologically, these cells resemble large granular lymphocytes, and typically express CD2, cytoplasmic CD3ϵ, CD56 and cytotoxic molecules, including perforin, granzyme B and TIA1, while lacking surface CD3 (**Figure 1**). This study was reviewed, approved, and performed in compliance with the ethical standards of the ethics committee of Beijing Tongren Hospital (Approval No. TRECKY2015-016), and the informed consent forms were obtained from each participant. The collected clinical data primarily included gender, age, B symptoms, Eastern Cooperative Oncology Group (ECOG) physical state score, whole blood cell analysis, lactate dehydrogenase (LDH) levels, enhanced Magnetic Resonance Imaging (MRI) of the paranasal sinus, computed tomography (CT) of chest and abdomen, positron emission tomography/computed tomography (PET/CT) of the whole body, bone marrow smear and biopsy, treatment regimens, patients’ responses, and toxicity assessment. Ann Arbor staging was applied for clinical staging classification, and the prognosis was evaluated according to the International Prognostic Index (IPI), Korean Prognostic Index (KPI), and Prognostic Index of Natural Killer Cell Lymphoma (PINK) scoring standards. The comprehensive evaluation of treatment efficacy adhered to the revised response criteria for malignant lymphoma established in 2007 (9). Within these criteria, CR indicates the disappearance of all evidence of disease, while PR reveals regression of measurable disease with no evidence of new sites.

### Treatments

Patients with ENKTL in stage I/II underwent the sandwich regimen, which involved induction chemotherapy followed by involved field radiotherapy (IFRT) and consolidation chemotherapy. Those who achieved complete remission (CR) or partial remission (PR) after 2 to 3 cycles of induction chemotherapy received sequential IFRT, with doses ranging from 50 to 55 Gy. For the consolidation chemotherapy, the same regimens were administered one month after radiotherapy. Patients with ENKTL in stage III/IV primarily underwent chemotherapy with or without autologous hematopoietic stem cell transplantation, and those with limited lesions after induction chemotherapy further received IFRT.

Patients diagnosed with ENKTL were classified into three groups, with each receiving a distinct first-line chemotherapy regimen. The first group (EPOCH-L) was treated with intravenous (IV) etoposide (50 mg/m^2^ on days 1 to 4), vindesine (1 mg on days 1 to 4, IV), epirubicin (15 mg/m^2^ on days 1 to 4, IV), dexamethasone (15 mg on days 1 to 5, IV), cyclophosphamide (750 mg/m^2^ on day 5, IV) and intramuscular (IM) L-asparaginase (10,000 U/day on days 6 to 10) or pegaspargase (2,500 U/m^2^ on day 2, IM). The second group (PD-Gemox) received gemcitabine (1.2 g/m^2^ on day 1, IV), oxaliplatin (130 mg/m^2^ on day 1, IV), pegaspargase (2,500 U/m^2^ on day 2, IM) and dexamethasone (20 mg on days 1 to 5, IV). The third group (SVILE) was administered ifosfamide (1 g/m^2^ on days 1 to 3, IV), pegaspargase (2,500 U/m^2^ on day 2, IM), vindesine (4 mg on day 1, IV), etoposide (75 mg/m^2^ on days 1 to 3, IV) and dexamethasone (20 mg on days 1 to 4, IV).

### Evaluation of the curative effects

Sinus enhancement MRI or CT was utilized to assess the local treatment efficacy, while contrast-enhanced CT or PET/CT was employed for a comprehensive whole-body evaluation. A post-chemotherapy assessment was performed after 2 to 3 cycles of chemotherapy but prior to radiotherapy, and the final evaluation was conducted 6 to 8 weeks after the completion of all treatments. The proportion of patients achieving CR or PR was calculated based on the overall response rate. Follow-up methods included hospitalization, outpatient re-examination, and phone surveys. The patients were followed up until February 2021, with a median follow-up duration of 48.9 months. Overall survival (OS) was defined as the duration from the date of first diagnosis to death from any cause or the end of the follow-up period. Progression-free survival (PFS) referred to the time from the date of first diagnosis to the initiation of disease progression or termination of the follow-up.

### Statistical analysis

R software (v. 4.0.3; The R Foundation, Vienna, Austria) was applied for statistical analysis. The Kaplan–Meier curves were employed for survival analysis, with survival comparisons conducted using the log-rank test. Univariate and multivariate Cox regression models were introduced for multivariate analysis of factors influencing prognosis. Statistical significance was set at P<0.05.

### Patients and public involvement

No patients or members of the public were involved in the study design, implementation of the study, measurement of outcomes, or interpretation of results.

## Results

**Figure 2:**
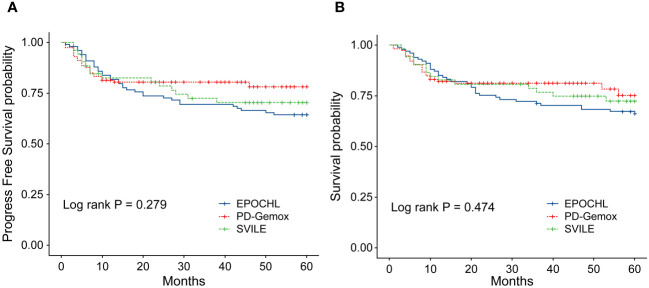
**(A)** Progression-free survival (PFS) of the 266 patients with ENKTL stratified by different treatment regimens. **(B)** Overall survival (OS) of the 266 ENKTL patients stratified by different treatment regimens.

The clinical features of the 266 patients are presented in **Table 1**. In general, among these patients, the male-to-female ratio was 2.13/1, and the median age was 47 years (ranging from 16 to 78). According to the Ann Arbor staging criteria, 198 patients were classified into stage I/II (74.44%), while 68 patients belonged to stage III/IV (25.56%). Moreover, 77 patients displayed elevated LDH levels (28.95%), and 193 patients combined with B symptoms (72.56%) at the time of diagnosis. Additionally, 217 patients (81.58%) presented with IPI scores in the range of 0 to 1, while 49 patients (18.42%) fell within the IPI scores of 2 to 5. The KPI score distribution was as follows: 0 for 47 patients (17.67%), 1 for 81 (30.45%), 2 for 75 (28.20%), and ≥3 for 63 (23.68%). All patients were evaluated by the PINK scoring system, with 163 patients (61.28%) scoring 0, 47 (17.67%) scoring 1, and 56 (21.05%) scoring ≥2. Furthermore, 20 patients (7.5%) underwent autologous hematopoietic stem cell transplantation. Notably, no statistical difference was observed in terms of clinical indices including gender, age, stages, IPI, KPI, PINK scoring systems, etc., and treatment outcomes among the three treatment groups (EPOCH-L, PD-Gemox, and SVILE) (**Table 1**, **Figures 2A, B**).

**Table 1:** Characteristics of 266 patients with ENKTL treated with three chemotherapy regimens.

	Total (n=266)	EPOCH-L (n=101)	PD-Gemox (n=113)	SVILE (n=52)	P
**Gender**					0.23
M	184 (69.17)	76 (75.25)	73 (64.60)	35 (67.31)	
F	82 (30.83)	25 (24.75)	40 (35.40)	17 (32.69)	
**Age**					0.12
≤60	218 (81.95)	90 (89.11)	88 (77.88)	40 (76.92)	
>60	48 (18.04)	11 (10.89)	25 (22.12)	12 (23.07)	
**Stage**					0.44
I/II	198 (74.44)	72 (71.29)	84 (74.34)	42 (80.77)	
III/IV	68 (25.56)	29 (28.71)	29 (25.66)	10 (19.23)	
**B symptoms**					0.41
Absent	73 (27.44)	27 (26.73)	35 (30.97)	11 (21.15)	
Present	193 (72.56)	74 (73.27)	78 (69.03)	41 (78.85)	
**LDH**					0.20
Normal	189 (71.05)	71 (70.30)	76 (67.26)	42 (80.77)	
Increased	77 (28.95)	30 (29.70)	37 (32.74)	10 (19.23)	
**ECOG**					0.46
0-1	243 (91.35)	90 (89.11)	106 (93.81)	47 (90.38)	
≥2	23 (8.65)	11 (10.89)	7 (6.19)	5 (9.62)	
**KPI**					0.44
0	47 (17.67)	14 (13.86)	22 (19.47)	11 (21.15)	
1	81 (30.45)	33 (32.67)	29 (25.66)	19 (36.54)	
2	75 (28.20)	26 (25.74)	37 (32.74)	12 (23.08)	
≥3	63 (23.68)	28 (27.72)	25 (22.12)	10 (19.23)	
**PINK**					0.16
Low	163 (61.28)	60 (59.41)	64 (56.64)	39 (75.00)	
Middle	47 (17.67)	16 (15.84)	24 (21.24)	7 (13.46)	
High	56 (21.05)	25 (24.75)	25 (22.12)	6 (11.54)	
**IPI**					0.57
0-1	217 (81.58)	82 (81.19)	90 (79.65)	45 (86.54)	
2-5	49 (18.42)	19 (18.81)	23 (20.35)	7 (13.46)	

ECOG, Eastern Cooperative Oncology Group; IPI, International Prognostic Index; KPI, Korean Prognostic Index; LDH, lactate dehydrogenase; PINK, Prognostic Index of Natural Killer Cell Lymphoma.

### Treatment schedule and evaluation of therapeutic outcomes

**Figure 3:**
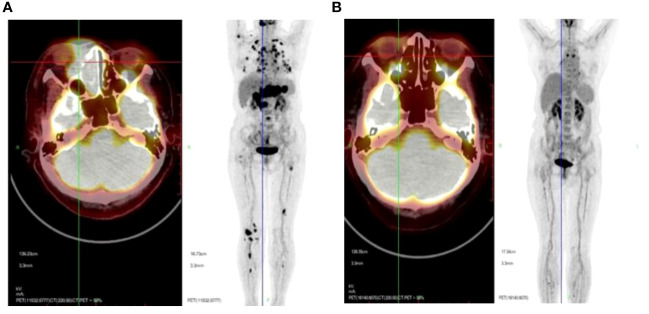
Representative PET/CT scans presented to offer a comparative evaluation of a patient diagnosed with stage IV ENKTL before **(A)** and after **(B)** treatment, with the assessment of therapeutic outcome as CR.

All 266 patients underwent at least four cycles of chemotherapy, and those in stage I/II also received radiotherapy. Among them, 189 patients achieved CR (71.5%), 10 reached PR (3.76%), and 67 experienced progressive disease (PD; 25.19%). The outcomes for the three different chemotherapy regimens are presented in **Table 2**. The PET/CT scans provided a comparative evaluation of a patient diagnosed with stage IV ENKTL before and after treatment, demonstrating the assessment of therapeutic outcome as CR (**Figures 3A, B**).

**Table 2:** The response rates for the different treatment regimens.

	Total (n=266)	EPOCH-L (n=101)	PD-Gemox (n=113)	SVILE (n=52)	P
					0.08
CR	189 (71.05)	63 (62.38)	89 (78.76)	37 (71.15)	
PR	10 (3.76)	5 (4.95)	2 (1.77)	3 (5.77)	
PD	67 (25.19)	33 (32.67)	22 (19.47)	12 (23.08)	

CR, complete remission; PD, Progressive Disease; PR, partial remission.

### EPOCH-L group

A total of 101 patients were treated with this regimen, including 72 patients (71.29%) in stage I/II and 29 (28.71%) in stage III/IV. After treatment, the CR rate was 62.38%, the PR rate was 4.95%, and 33 patients (32.67%) exhibited PD.

### PD-Gemox group

A total of 113 patients received this regimen, including 84 patients (74.34%) in stage I/II and 29 (25.66%) in stage III/IV. After treatment, the CR rate was 78.76%, the PR rate was 1.77%, and 22 patients (19.47%) experienced PD.

### SVILE group

A total of 52 patients underwent this regimen, with 42 patients (80.77%) in stage I/II and 10 (19.23%) in stage III/IV. After treatment, the CR, PR and PD rates were 71.15%, 5.77% and 23.08%, respectively.

### Long-term survival

**Figure 4:**
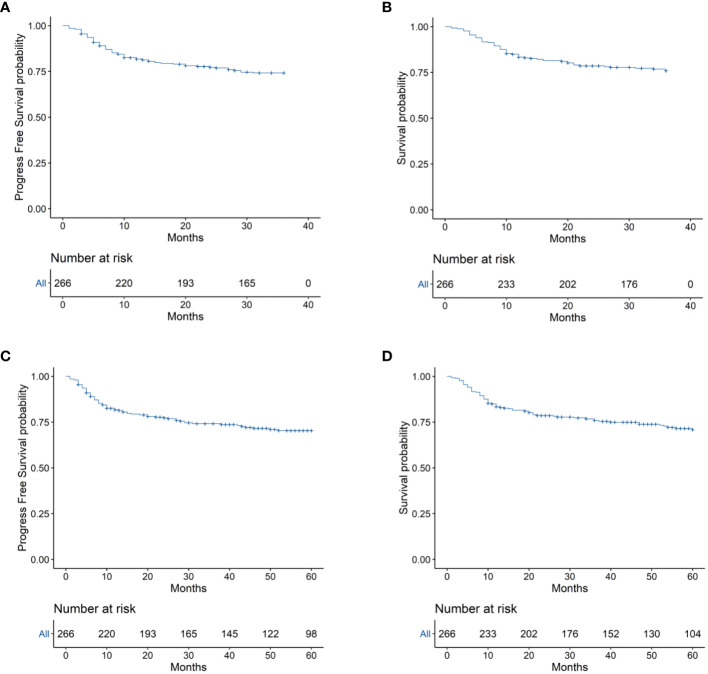
**(A)** 3-year Progression-free survival (PFS) of the 266 patients with ENKTL. **(B)** 3-year Overall survival (OS) of the 266 ENKTL patients. **(C)** 5-year Progression-free survival (PFS) of the 266 patients with ENKTL. **(D)** 5-year Overall survival (OS) of the 266 ENKTL patients.

**Figure 5:**
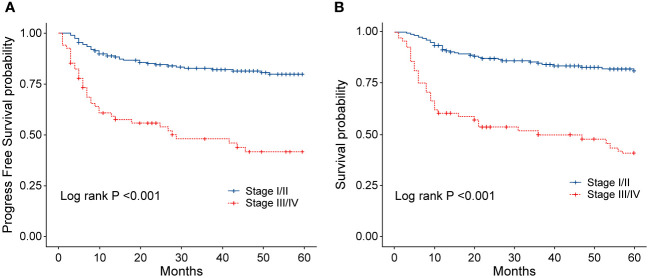
**(A)** Progression-free survival (PFS) of patients with ENKTL in different stages. **(B)** Overall survival (OS) of patients with ENKTL in different stages.

To evaluate the long-term prognosis after treatment, the PFS and OS of the patients were included in the analysis. In this study, a total of 68 deaths were recorded, with 31 in the EPOCH-L group, 14 in the SVILE group, and 23 in the PD-Gemox group. Among these, 64 deaths were attributed to ENKTL disease progression, two were associated with the adverse effects of the treatment, and the other two were unrelated to this disease. For all patients, the median follow-up time was 48.9 months. The PFS rates at 3 and 5 years were 74.2% and 70.4%, respectively, while the OS rates at 3 and 5 years were 75.9% and 70.9%, respectively (**Figures 4A–D**). Moreover, the 3-year and 5-year PFS rates of patients in stage I/II were 82.7% and 79.8%, respectively, while the OS rates of these patients were 84.8% and 81.1%, respectively. For the patients in stage III/IV, the 3-year and 5-year PFS rates were 48.1% and 41.7%, respectively, while the OS rates were 49.8% and 40.9%, respectively (**Figures 5A, B**). No statistical difference regarding the long-term outcomes among patients treated with the three different chemotherapy regimens was observed.

### Analysis of factors influencing the prognosis of patients

**Figure 6:**
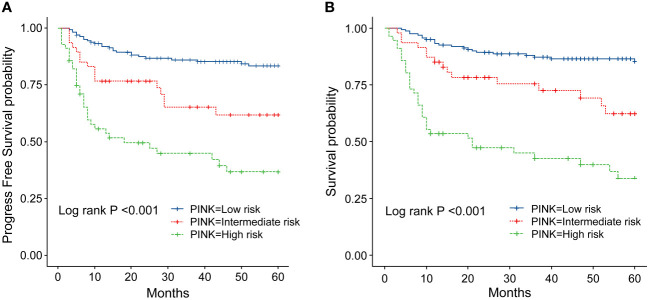
**(A)** Progression-survival (PFS) of ENKTL patients with different PINK scores. **(B)** Overall survival (OS) of patients with ENKTL with different PINK scores.

**Figure 7:**
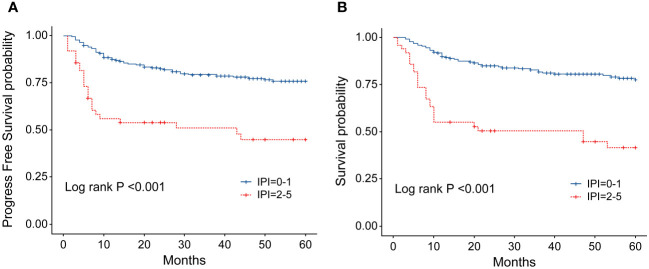
**(A)** Progression-free survival (PFS) of patients with ENKTL with different IPI scores. **(B)** Overall survival (OS) of patients with ENKTL with different IPI scores.

**Figure 8:**
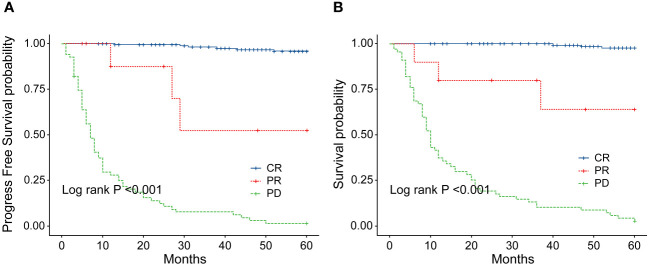
**(A)** Comparison of progression-free survival (PFS) between patients with ENKTL who achieve complete response (CR) and those who did not after 2–4 cycles of chemotherapy. **(B)** Comparison of overall survival (OS) between patients with ENKTL who achieve complete response (CR) and those who did not after 2–4 cycles of chemotherapy.

**Figure 9:**
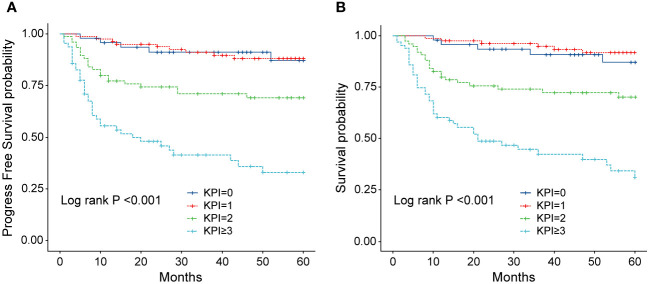
**(A)** Progression-free survival (PFS) of patients with ENKTL with different KPI scores. **(B)** Overall survival (OS) of patients with ENKTL with different KPI scores.

In order to identify the factors influencing prognosis, we conducted univariate survival analysis on the 266 patients. The results demonstrated that adverse prognostic factors affecting both PFS and OS included Ann Arbor stage, ECOG score, elevated LDH, IPI score, PINK score, KPI score, and whether CR was achieved after all treatment courses (**Figures 6**–**9**). However, age, gender, B symptoms, and the type of chemotherapy regimen used in the patients had no significant influence on PFS and OS. A Cox regression model was applied for multivariate analysis, revealing that the achievement of CR immediately after treatment was considered as the sole independent adverse prognostic factor for both PFS and OS in patients with ENKTL (**Table 3**).

**Table 3:** Univariate and multivariate analysis of prognostic factors for survival (by Cox Regression).

	PFS				OS			
	Univariate		Multivariate		Univariate		Multivariate	
	P	HR (95% CI)	P	HR (95% CI)	P	HR( 95% CI)	P	HR (95% CI)
Age >60	0.08	1.011 (0.995–1.028)	–	–	0.05	1.796 (1.015–3.176)	–	–
Gender	0.59	0.872 (0.528–1.439)	–	–	0.39	0.797 (0.472–1.345)	–	–
ECOG ≥2	<0.001	10.708 (6.185–18.536)	–	–	<0.001	12.150 (7.112–20.758)		
Stage III/IV	<0.001	3.738 (2.391–5.842)	0.424	0.705 (0.298–1.663)	<0.001	4.275 (2.697–6.776)	0.10	0.736 (0.303,1.791)
Elevated LDH	0.05	1.596 (1.004–2.537)	–	–	0.02	1.742 (1.086–2.792)	–	–
CR after treatment	<0.001	15.735 (3.917–63.205)	<0.001	12.111 (2.925–50.139)	<0.001	28.012 (5.602, 140.063)	<0.001	24.059 (4.656-124.333)
B syndrome	0.92	0.975 (0.596–1.597)	–	–	0.61	0.879 (0.533–1.450)	–	–
IPI 2-5	<0.001	3.036 (1.884– 4.895)	–	–	<0.001	3.766 (2.336– 6.069)	–	–
KPI	<0.001	2.244 (1.828–2.755)	–	–	<0.001	2.436 (1.967–3.016)	–	–
PINK	<0.001	2.349 (1.851–2.981)	–		<0.001	2.605 (2.033–3.337)	–	–
Treated EPOCH-L PD-Gemox SVILE	Ref 0.09 0.52	0.635 (0.376–1.070) 0.819 (0.449–1.495)	–	–	Ref 0.20 0.48	0.705 (0.416–1.196) 0.799 (0.430–1.484)	–	–

CI, confidence interval; CR, complete remission; ECOG, Eastern Cooperative Oncology Group; HR, hazard ratio; IPI, International Prognostic Index; KPI, Korean Prognostic Index; LDH, lactate dehydrogenase; OS, overall survival; PFS, progression-free survival; PINK, Prognostic Index of Natural Killer Cell Lymphoma; Ref, reference.

### Adverse effects

All adverse reactions resulting from treatments using the three different chemotherapy schemes were summarized in **Table 4**. The most common adverse event was hematological toxicity. Grade 3/4 neutropenia occurred in 71 patients (70.3%) in the EPOCH-L group, 46 patients (40.7%) in the PD-Gemox group, and 29 patients (55.7%) in the SVILE group. Additionally, 34 patients (33.6%) in the EPOCH-L group, 27 patients (23.8%) in the PD-Gemox group, and 9 patients (17.3%) in the SVILE group experienced Grade 3/4 thrombocytopenia. However, no statistical difference was observed among the three treatment groups. Grade 3/4 liver dysfunction appeared in 8 patients (7.9%) in the EPOCH-L group, 8 patients (7.0%) in the PD-Gemox group, and 3 patients (5.7%) in the SVILE group. No statistical difference was observed among the groups in terms of non-hematological toxicity (**Table 4**).

**Table 4:** Grade 3/4 toxicity that occurred in the three treatment groups.

Toxicity	EPOCH-L (n=101)	PD-Gemox (n=113)	SVILE (n=52)	P
Hematologic toxicity				
Leukopenia	47 (46.5)	29(25.6)	19(36.5)	0.24
Neutropenia	71 (70.3)	46(40.7)	29(55.7)	0.15
Anemia	17 (17.8)	22(19.5)	10(19.2)	0.93
Thrombocytopenia	34 (33.6)	27(23.8)	9(17.3)	0.53
Gastrointestinal disorders	13 (12.8)	13(11.6)	5(9.6)	0.83
Coagulopathy				
APTT elongation	7 (6.9)	8(7.1)	3(5.8)	0.97
Hypofibrinogenemia	13 (12.9)	21(18.5)	11(21.1)	0.57
Liver dysfunction	8 (7.9)	8(7.0)	3(5.7)	0.42
Increase in BUN	0	0	0	0.66

APTT, activated partial thromboplastin time; BUN, blood urea nitrogen.

## Discussion

ENKTL is a peripheral NK/T-cell lymphoma originating in the nasal cavity or other extranodal organs, and its pathogenesis is tightly associated with EB virus infection (10). Histologically, it is characterized by central vascular infiltration, tissue necrosis, and expression of NK/T-cell markers. Clinically, ENKTL is aggressive and typically manifests in the facial midline structure with destructive lesions. The geographical distribution of this disease is race-specific, commonly occurring in East Asia and Latin America. A collaborative study conducted by International T-cell Lymphoma Project Group identified 1,314 patients with peripheral T-cell lymphoma, with ENKTL accounting for 5.1% in North America, 4.3% in Europe, and 22.4% in Asia (11). The Chinese Lymphoma Pathology Research Cooperation Group analyzed 10,002 lymphoma cases, in which ENKTL accounted for 6.02% of non-Hodgkin’s lymphoma and 28% of T-cell lymphoma cases, suggesting that this lymphoma subtype is more prevalent in China compared to other countries (12).

Most ENKTLs manifest in the upper respiratory tract and exhibit similar prognosis irrespective of whether the primary location is the nasal cavity, the nasopharynx, or Wechsler’s ring. Upper aerodigestive tract (UAT)-NKTL is a subtype of lymphoma occurring in the upper respiratory and digestive tracts. ENKTLs originating from non-upper respiratory and digestive tracts are known as N-UAT-NKTL, representing a relatively rare subtype that occurs in the skin, testicles, or the gastrointestinal tract (13). N-UAT-NKTL is associated with multiple adverse prognostic factors, and the reported median OS for N-UAT-NKTL is only 3 months, indicating a particularly aggressive nature of this subtype (2, 14). In our study, 266 patients had ENKTL manifesting in the upper respiratory and digestive tract, originating from the nasal cavity, nasopharynx, and oropharynx, as well as adjacent structures such as the sinus, orbit, surrounding bone and facial skin. The Ann Arbor staging system for ENKTL classification was applied under this condition. It is reported that 70 to 90% of ENKTL patients are in stage I/II and 10 to 30% are in stage III/IV at the initial diagnosis (15), whereas in our study, 198 patients with stage I/II disease constituted 74.44%, while 68 patients with stage III/IV disease accounted for 25.56% among the entire participants. However, given that ENKTL often originates from extranodal sites, is frequently associated with local invasion, and distant involvement is not commonly observed, the Ann Arbor staging system exhibits certain limitations. Nevertheless, due to the absence of a more effective staging system, all current treatments are still based on it.

At present, patients in the early stages are treated with chemotherapy combined with radiotherapy, particularly for those with risk factors including B symptoms, elevated LDH levels, and hyperactivity of the involved tissues (16). Conversely, radiotherapy or chemotherapy alone depicts a high risk of progression and recurrence (17). Common treatments include sequential or sandwich radiotherapy and chemotherapy after induction, as well as sequential chemotherapy following radiotherapy. For patients with advanced and refractory recurrence, comprehensive chemotherapy is the primary treatment choice. In the selection of first-line chemotherapy regimens, ENKTL is insensitive to anthracycline-containing treatment due to the high expression of P-glycoprotein in the tumor cells, resulting in multidrug resistance (18). Recently, levoasparagase or peasparagase has become the main drug in the treatment regimens and has been widely used in clinical settings to achieve favorable therapeutic effects.

The China Lymphoma Collaborative Group (CLCG) reported the follow-up data from 2,560 patients with ENKTL in 20 hospitals in China, of which 68.7% were in a limited stage. According to the report, with a median follow-up of 4 years, the CR rate after treatment with non-anthracycline drugs was 45%, and the 5-year OS rate was 68.9%. In contrast, the CR rate in the anthracycline chemotherapy group was only 28%, with a 5-year OS rate of 57.5% (19, 20). The application of non-anthracycline chemotherapy regimens containing asparaginase significantly improved the therapeutic outcomes for the patients with limited-stage disease, achieving a 5-year OS of 70 to 80% and PFS above 60%. In addition, the role of radiotherapy remains irreplaceable. The majority of investigations concerning the sequence of chemotherapy and radiotherapy were retrospective analyses, and they indicated no significant difference in this regard (17, 21). In this study, sandwich therapy combining radiotherapy and chemotherapy was employed for patients with stage I/II ENKTL. After treatment, the OS at 3 and 5 years was 84.8% and 81.1%, respectively, and this high response rate is consistent with those reported by other centers.

Despite the application of the regimen containing asparaginase for chemotherapy in patients with advanced disease, the OS rate at 5 years was approximately 30%, with a median OS of only 6 to 12 months. In this study, the OS rates for the 68 patients with stage III/IV ENKTL at 3 and 5 years were 49.8% and 40.9%, respectively, which were significantly lower than those for patients in stage I/II, indicating an unfavorable prognosis among the patients with advanced disease. Currently, the most commonly used schemes for the treatment of ENKTL include SMILE, DDGP and P-Gemox, with particular emphasis on the application of the SMILE regimen in advanced ENKTL. This regimen has demonstrated a relatively good therapeutic effect, with an Overall Response Rate (ORR) rate ranging from 70% to 80% (22). However, a major concern was the treatment-related toxicity, especially for the Asian population. Recent studies indicated that 100% of patients who underwent this treatment experienced Grade 3 to 4 hematological toxicity, and the infection rate was as high as 45%. Therefore, the SMILE scheme may not be considered ideal for the treatment of patients with stage III/IV ENKTL (23–25). Based on these results, our center replaced methotrexate with vindesine in the SVILE regimen (52 cases), which revealed a better response rate than that reported in the previous clinical research. The OR rates of patients in stage I/II and stage III/IV were approximately 90% and 75%, respectively, and the toxicity was tolerable (26).

Other gemcitabine-containing regimens including the DDGP or P-Gemox, are currently being applied in many medical centers, demonstrating comparable efficacy rates. Moreover, the treatment-related toxicity is manageable, with a mortality rate at 9 to 10%. In this study, 266 patients were treated with chemotherapy regimens including asparaginase. Analysis of CR, ORR, OS, and PFS rates at 3 and 5 years across the three distinct chemotherapy schemes revealed no statistically significant differences. We speculated that the incorporation of asparaginase into various chemotherapy combinations improved therapeutic effects with manageable treatment-related toxicity, primarily hematological toxicity.

The IPI scoring system holds significant prognostic value for non-Hodgkin’s lymphoma, and is also applicable to ENKTL. However, the utility of this scoring system is constrained by the extranodal onset of ENKTL, extensive local involvement, and limited distant invasion (27). Addressing the shortcomings of the KPI scoring system, a multicenter retrospective clinical study in Korea included B symptoms and elevated LDH, yet failed to sufficiently stratify ENKTL prognosis (28). Regarding non-anthracycline chemotherapy, PINK and PINK-E models demonstrated superior efficacy in risk stratification for moderate-risk patients based on the IPI scoring system. One study systematically analyzed 527 patients, identifying age over 60 years, stage III/IV lesions, distant lymph node involvement, non-nasal type, and elevated EB virus DNA copy number as critical factors for PFS and OS. In addition, the ENKTL nomogram and nomogram-revised risk index (NRI) model, derived from extensive Chinese data, offered improved individual prediction of OS, especially in the risk stratification of early-stage ENKTL (29, 30). In our study, we employed the IPI, KPI, and PINK scoring systems to classify patients into different risk groups and analyzed their survival outcomes. While the IPI and KPI systems failed to effectively differentiate between low- and moderate-risk groups, the PINK scoring system demonstrated enhanced discriminatory ability. For instance, using the KPI system, the 5-year OS rates for low- and moderate-risk patients were 90.2% and 87.6%, respectively. Conversely, when employing the PINK scoring system, the corresponding 5-year OS rates for low-, moderate-, and high-risk patients were 89.2%, 68.3%, and 37.5%, respectively. Furthermore, survival analysis revealed that an ECOG performance status of ≥2, stage III/IV lesions, LDH elevation, and achieving CR after treatment were adverse factors affecting PFS and OS. However, in multivariate analysis, none of these adverse factors demonstrated independent prognostic significance, expect for immediate CR attainment post-treatment.

At present, chemotherapy remains the cornerstone of treatment for ENKTL, particularly with the addition of a regimen containing asparaginase to the first-line chemotherapy. The results of our study illustrated that chemotherapy regimens containing asparaginase significantly improved the survival of patients with ENKTL, as indicated by the 5-year OS rate for those with limited lesions in stage I/II achieving 81.1%. In contrast, the prognosis of patients in stage III/IV is unfavorable, with their 5-year OS rate only reaching 40.9%. Currently, it is crucial to improve the cure rate of patients with advanced ENKTL and prolong their OS. The incorporation of new treatment modalities, including monoclonal antibodies, immune checkpoint inhibitors, and immunomodulators, has been reported to play an effective role in refractory and recurrent patients with ENKTL, leading to an increase in the remission rate. This will be the focus of our future research.

Regarding prognosis, many of the prognostic scoring systems currently utilized have limitations. This study revealed that an ECOG performance status ≥2, stage III/IV lesions, and LDH elevation influence prognosis; however, whether patients achieve CR after treatment emerges as an independent adverse factor for the prognosis of patients with ENKTL. Nonetheless, many limitations and deficiencies exist in our study, including the absence of the clinical profile of patients regarding EB virus infection status, which is an important index for evaluating the outcomes of patients, and omission of certain statistics and analyses. These aspects will be addressed and further refined in our subsequent investigations.

## Data availability statement

The original contributions presented in the study are included in the article/supplementary material. Further inquiries can be directed to the corresponding authors.

## Ethics statement

The studies involving humans were approved by Ethics Committee of Beijing Tongren Hospital. The studies were conducted in accordance with the local legislation and institutional requirements. The participants provided their written informed consent to participate in this study.

## Author contributions

LY: Writing – original draft. JW: Writing – review & editing. LQW: Writing – original draft. XL: Writing – original draft. JC: Writing – original draft. JYe: Writing – original draft. NY: Writing – original draft. JYa: Writing – original draft. LW: Supervision, Funding acquisition, Writing – review & editing.
